# OSI-027 inhibits pancreatic ductal adenocarcinoma cell proliferation and enhances the therapeutic effect of gemcitabine both *in vitro* and *in vivo*

**DOI:** 10.18632/oncotarget.4579

**Published:** 2015-07-21

**Authors:** Xiao Zhi, Wei Chen, Fei Xue, Chao Liang, Bryan Wei Chen, Yue Zhou, Liang Wen, Liqiang Hu, Jian Shen, Xueli Bai, Tingbo Liang

**Affiliations:** ^1^ Department of Hepatobiliary and Pancreatic Surgery, The Second Affiliated Hospital, School of Medicine, Zhejiang University, Hangzhou, P.R.China; ^2^ Key Laboratory of Cancer Prevention and Intervention, The Second Affiliated Hospital, Zhejiang University School of Medicine, Hangzhou, P.R.China; ^3^ Collaborative Innovation Center for Cancer Medicine, Zhejiang University, Hangzhou, P.R.China

**Keywords:** PDAC, mTOR, OSI-027, gemcitabine, multidrug resistance

## Abstract

Despite its relative rarity, pancreatic ductal adenocarcinoma (PDAC) accounts for a large percentage of cancer deaths. In this study, we investigated the *in vitro* efficacy of OSI-027, a selective inhibitor of mammalian target of rapamycin complex 1 (mTORC1) and mTORC2, to treat PDAC cell lines alone, and in combination with gemcitabine (GEM). Similarly, we tested the efficacy of these two compounds in a xenograft mouse model of PDAC. OSI-027 significantly arrested cell cycle in G0/G1 phase, inhibited the proliferation of Panc-1, BxPC-3, and CFPAC-1 cells, and downregulated mTORC1, mTORC2, phospho-Akt, phospho-p70S6K, phospho-4E-BP1, cyclin D1, and cyclin-dependent kinase 4 (CDK4) in these cells. Moreover, OSI-027 also downregulated multidrug resistance (MDR)-1, which has been implicated in chemotherapy resistance in PDAC cells and enhanced apoptosis induced by GEM in the three PDAC cell lines. When combined, OSI-027 with GEM showed synergistic cytotoxic effects both *in vitro* and *in vivo*. This is the first evidence of the efficacy of OSI-027 in PDAC and may provide the groundwork for a new clinical PDAC therapy.

## INTRODUCTION

Pancreatic ductal adenocarcinoma (PDAC) is the twelfth most common cancer worldwide, with an incidence of 6 to 8 people per 100, 000 males in developed countries [1, 2]. However, despite its relatively low incidence, PDAC is the fourth most deadly cancer [3], and more than 95% of patients are expected to die from it in five years [4], which is a consequence of its asymptomatic nature and thus it is often diagnostic after it is well-advanced [2].

Although surgical resection is the preferred treatment for PDAC, the advanced stage at diagnosis renders most tumors inoperable. Thus, there has been much research investigating novel treatments for this disease. Fluorouracil (5-FU) was traditionally used as first-line chemotherapy, but gemcitabine (GEM), another nucleoside analogue, has shown greater efficacy and has usurped the former as the standard chemotherapy [5]. However, even with GEM treatment, 5-year survival rates from PDAC remain still low at just over 20% [6].

The mammalian target of rapamycin (mTOR) is an atypical serine/threonine kinase that amalgamates many different cellular processes, including cell growth and metabolism [7, 8]. mTOR pathways are often aberrantly activated in a wide variety of tumors, and the hyperactivated mTOR pathways contribute to tumor proliferation and survival [9, 10, 11, 12]. mTOR consists of two complexes, mTOR complex (C) 1, and mTORC2, the former being implicated in protein synthesis [13, 14, 15], while the latter regulates the cytoskeleton [13, 14, 15]. mTORC1 forms a complex with various proteins, mainly regulatory-associated protein of mTOR (Raptor), while mTORC2 combines with rapamycin-insensitive companion of mTOR (Rictor). Rapamycin (RAPA), the drug of the mTOR namesake, inhibits mTORC1 and has shown some promise for treating metastatic renal cancer [16], prostate cancer [17], and PDAC [18, 19]. However, it does not suppress mTORC2. Recent studies have shown that inhibit mTORC1 and mTORC2 is advantageous for treating leukemia and other cancers [20, 21].

OSI-027 is an orally bioavailable, specific and potent ATP-competitive mTORC1 and mTORC2 inhibitor that is currently in phase II clinical trials. It has shown greater promise than RAPA in preclinical studies involving a variety of tumor types, such as Leukemia [22, 23], lymphomas [24], hepatocellular carcinoma [25], and colorectal cancer [26]. However, the effect of OSI-027 in PDAC remains unclear. Thus, in this study, the effects of OSI-027 were examined *in vitro* and in an *in vivo* PDAC model in combination with GEM.

## RESULTS

### The effects of OSI-027 and RAPA on PDAC cell line viability and cell cycle

**Figure 1:**
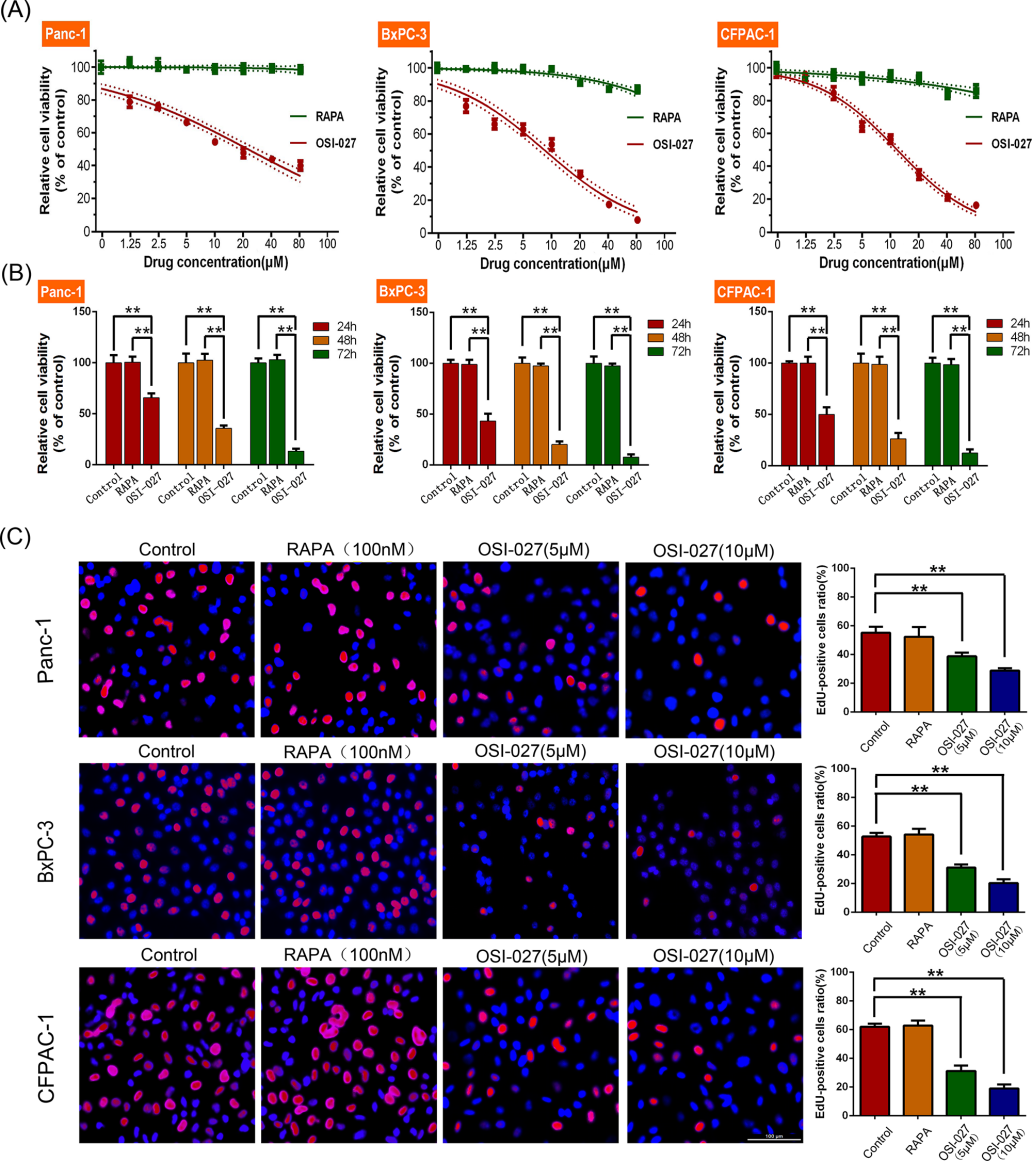
The effects of rapamycin (RAPA) and OSI-027 on cell viability and cell proliferation **A.** CCK-8 assay revealed that OSI-027 significantly reduced cell viability in PDAC cell lines (Panc-1, BxPC-3, and CFPAC-1) compared to RAPA (*P* < 0.05). **B.** The cytotoxicity of OSI-027 (10 μM) was time-dependent in PDAC cell lines (**P < 0.01). **C.** Cell-light EdU Apollo 488 *in vitro* kit assay showed OSI-027 inhibited DNA synthesis, whereas RAPA had little effect (**P < 0.01).

**Figure 2:**
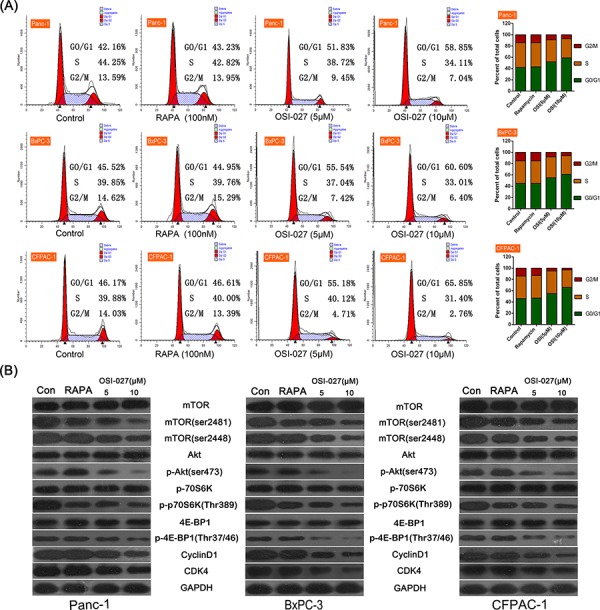
The effects of rapamycin (RAPA) and OSI-027 on the cell cycle of three PDAC cell lines (Panc-1, BxPC-3, CFPAC-1) **A.** Flow cytometry analysis of RAPA and two doses of OSI-027 (5 and 10 μM). Both doses of OSI-027 caused more cells to smiddle in the G0/G1 phase than RAPA. **B.** Western blot of different mTOR-related proteins in the three cell lines with OSI-027 (5 and 10 μM) and RAPA. OSI-027 (10 μM) significantly reduced mTORC2, phospho-Akt, cyclin D1, CDK4 compared to RAPA (*P* < 0.05).

OSI-027 significantly reduced cell viability in PDAC cell lines (Panc-1, BxPC-3, and CFPAC-1) compared to controls after 24 hours of treatment, whereas RAPA had little effect (*P* < 0.05; Figure 1A). The IC_50_ of OSI-027 is shown in Table 1. The cytotoxicity of OSI-027 (10 μM) was time-dependent and RAPA still had no effects on PDAC cells after 48 and 72 hours drug treatment (Figure 1B). Edu incorporation showed that OSI-027 also significantly inhibited DNA synthesis. Conversely, RAPA had little effect on the proliferation of these PDAC cells (*P* < 0.05; Figure 1C). Flow cytometry assay showed 5 μM OSI-027 induced cell cycle arrest in the G0/G1 phase in Panc-1, BxPC-3, and CFPAC-1 cells, whereas RAPA had little effect on cell cycle. Moreover, 10 μM OSI-027 further upregulated the proportion of cells arrested in G0/G1 phase (Figure 2A).

**Table 1:** IC50 values and statistical analyses of gemcitabine (GEM) and OSI-027 treatments in PDAC cell lines

Cell lines	IC50			
	GEM (μM)	OSI-027 (μM)	OSI-027 (μM) + GEM (μM)	Interaction
Panc-1	38.86(18.51 to 59.20)	21.57(16.27 to 26.87)	OSI 8.891(7.943 to 9.839) GEM 0.8891(0.7943 to 0.9839)	0.435 synergy
BxPC-3	1.156(0.9568 to 1.356)	8.360(7.511 to 9.206)	OSI 3.381(3.110 to 3.625) GEM 0.3381(0.3110 to 0.3625)	0.696 synergy
CFPAC-1	0.9702(0.893 to 1.047)	13.72(12.44 to 14.99)	OSI 2.751(2.588 to 2.915) GEM 0.2751(0.2588 to 0.2915)	0.484 synergy

### Changes in mTOR expression following OSI-027 treatment

To further investigate the underlying mechanism by which OSI-027 induced cell cycle arrest, we tested the changes of mTOR pathway proteins after treatment of OSI-027 and RAPA, Western blot analyses showed that both 5 μM and 10 μM OSI-027 inhibited phosphorylation of mTOR (ser2481), mTOR (ser2448), Akt, and downstream effectors of the mTOR pathway, including p-4E-BP-1 and p-p70S6K. Moreover, OSI-027 significantly downregulated the expression of Cyclin D1 and CDK4, which was important in the regulation of cell cycle. RAPA only inhibited mTOR (ser2481) and p70S6K phosphorylation (*P* < 0.05); however, it up-regulated p-Akt and had no significant effect on 4E-BP-1 phosphorylation, and the expression of Cyclin D1 and CDK4 (Figure 2B).

### Effects of Raptor and Rictor on cell cycle in PDAC cells

**Figure 3:**
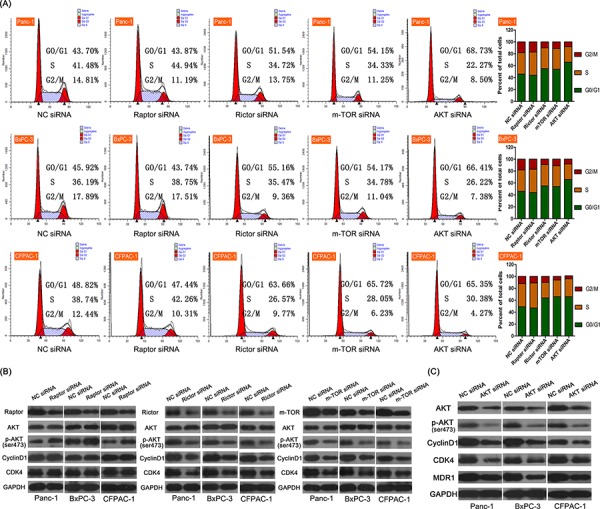
Effects of downregulating several key mTOR-related proteins using small interfering (si) RNA in three PDAC cell lines (Panc-1, BxPC-3, CFPAC-1) **A.** Flow cytometry analysis of the cell cycle of the three cell lines following non-coding (NC), Raptor, Rictor, mTOR, and Akt siRNA. Only Rictor, mTOR, and Akt significantly inhibited proliferation (*P* < 0.05). **B.** Western blot showing the effects of the same siRNA shown in (A) on various downstream effectors of mTOR. Rictor and mTOR siRNA depressed expression of most proteins. **C.** Akt siRNA decreased Cyclin D1, CDK4, and MDR1 expression.

In order to confirm the role of mTOR pathway in the regulation of cell cycle, we performed small interfering (si) RNA knock-out in Panc-1, BxPC-3, and CFPAC-1 cells, and found that siRNA application successfully knocked down the expression of mTOR, Raptor (mTOR1) and Rictor (mTOR2) in these cells. Cell cycle analyses showed that Raptor siRNA had no effect on the cell cycle; however, Rictor siRNA, total mTOR siRNA, and Akt siRNA all significantly increased the proportion of cells in the G0/G1 phase (Figure 3A), suggesting that inhibition mTOR2 in PDAC may be a viable approach to inhibit cell growth. Western blot analyses revealed that while Raptor siRNA increased Akt phosphorylation, it exerted no effect on Cyclin D1 or CDK4 expression (Figure 3B). Conversely, Rictor siRNA and mTOR siRNA both inhibited Akt phosphorylation and downregulated Cyclin D1 and CDK4 expression. When Akt was knocked out in the pancreatic three cell lines, the expression of Cyclin D1, CDK4, and MDR1 were all subsequently downregulated (Figure 3C).

### The effects of OSI-027 and GEM on PDAC cell lines

**Figure 4:**
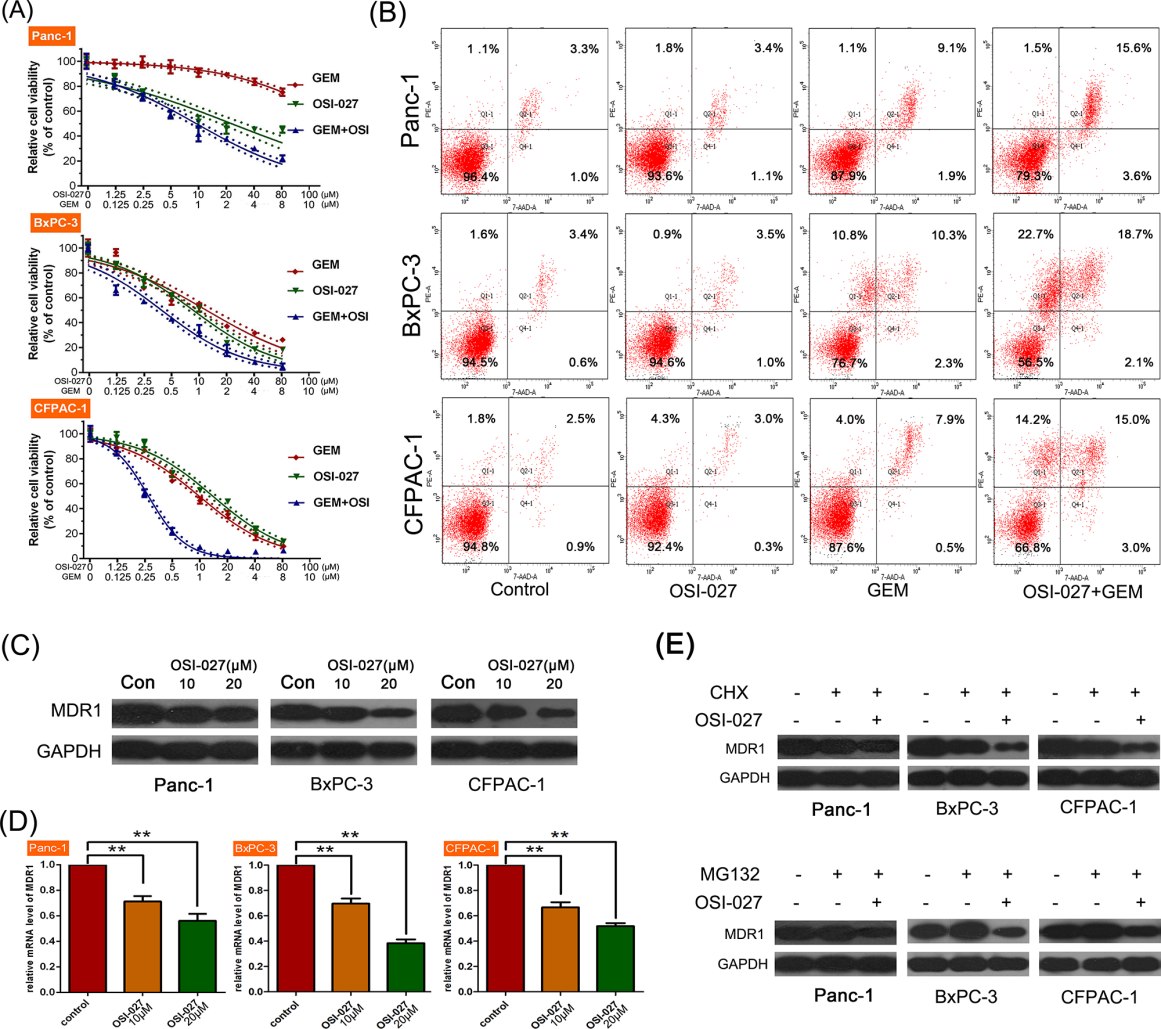
The effects of OSI-027 and gemcitabine (GEM) on three PDAC cell lines (Panc-1, BxPC-3, and CFPAC-1) **A.** CCK-8 analysis showing that the combination of OSI-027 and GEM was more cytotoxic than either compound alone. **B.** Flow cytometry analysis showing that OSI-027 could only enhance apoptosis when combined with GEM. **C.** Western blot and **D.** PCR analysis showing OSI-027 reduced MDR-1 expression at both the protein and RNA level (**P < 0.01). **E.** CHX with OSI-027 significantly reduced MDR1 expression. In addition, MG-132 alone increased MDR1 levels, but when combined with OSI-027, MDR1 levels were reduced relative to the control.

In the test of synergistic effect of OSI-027 and GEM, CCK-8 assay revealed that the combination of OSI-027 and GEM was more cytotoxic in all three cell lines compared to either treatment alone (Figure 4A). Moreover, CI analysis showed that combination treatment had a synergistic killing effect in all three cell lines (Table 1).

Apoptosis analysis revealed that OSI-027 could not induce cell apoptosis; however, when combined with GEM, it significantly enhanced apoptosis induced by GEM (Figure 4B).

### The effects of OSI-027 and combination therapy on MDR1

Western blot and PCR analyses revealed that MDR1 mRNA and the MDR1 protein were significantly downregulated following OSI-027 treatment (Figure 4C and 4D). OSI-027 enhanced the inhibition of MDR1 expression induced by CHX, a protein synthesis inhibitor, in Panc-1, BxPC and CFPCA-1. Moreover, OSI-027 reversed the upregulation of MDR1 expression induced by MG123, which is a protein degradation inhibitor (Figure 4E), suggesting that OSI-027 downregulated the expression of MDR-1 through inhibiting MDR-1 protein synthesis.

**Figure 5:**
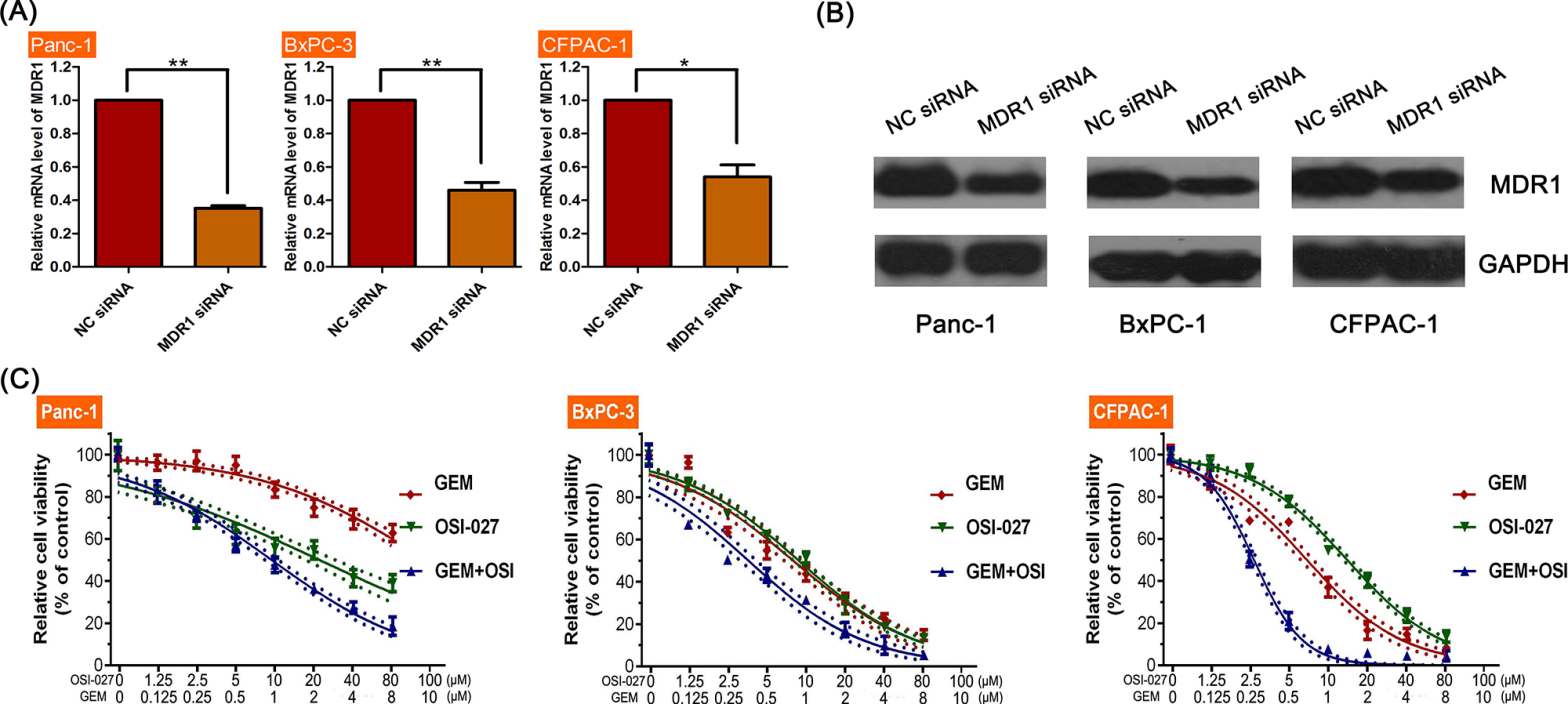
The efficacy of OSI-027 and gemcitabine (GEM) on PDAC cell line viability following MDR1 inhibition **A.** MDR-1 siRNA significantly reduced MDR-1 mRNA levels compared to controls in Panc-1, BxPC-3 (**P < 0.01), and CFPAC-1 (*P < 0.05). **B.** Western blot confirmation of MDR-1 protein inhibition following MDR-1 siRNA. **C.** MDR-1 siRNA inhibited the synergistic effects of GEM and OSI-027.

MDR1 siRNA inhibited the expression of both MDR1 mRNA and protein synthesis (Figure 5A, 5B). Moreover, the synergistic effect of GEM and OSI-027 combination treatment in all three PDAC cell lines were inhibited by MDR1 siRNA treatments (Figure 5C). The IC50 of GEM was significantly decreased in MDR1 siRNA treated cells (Table 2).

**Table 2:** MDR1 small interfering (si) RNA IC50 values and statistical analyses of gemcitabine (GEM) and OSI-027 treatments in PDAC cell lines

Cell lines	IC50			
	GEM (μM)	OSI-027 (μM)	OSI-027 (μM) + GEM (μM)	Interaction
Panc-1	14.79 (10.34 to 19.20)	21.93 (17.37 to 26.48)	OSI8.951 (8.309 to 9.594) GEM0.8951 (0.8309 to 0.9594)	0.465 synergy
BxPC-3	0.7716 (0.6574 to 1.356)	8.551 (7.913 to9.187)	OSI 3.041 (2.799 to9.594) GEM 0.3041 (0.279 to 0.959)	0.749 synergy
CFPAC-1	0.6796 (0.6082 to 0.751)	13.96 (13.06 to 14.85)	OSI 2.702 (2.554 to2.850) GEM0.2702 (0.2554 to 0.2850)	0.59 synergy

### The effects of OSI-027 and GEM in a mouse model

**Figure 6:**
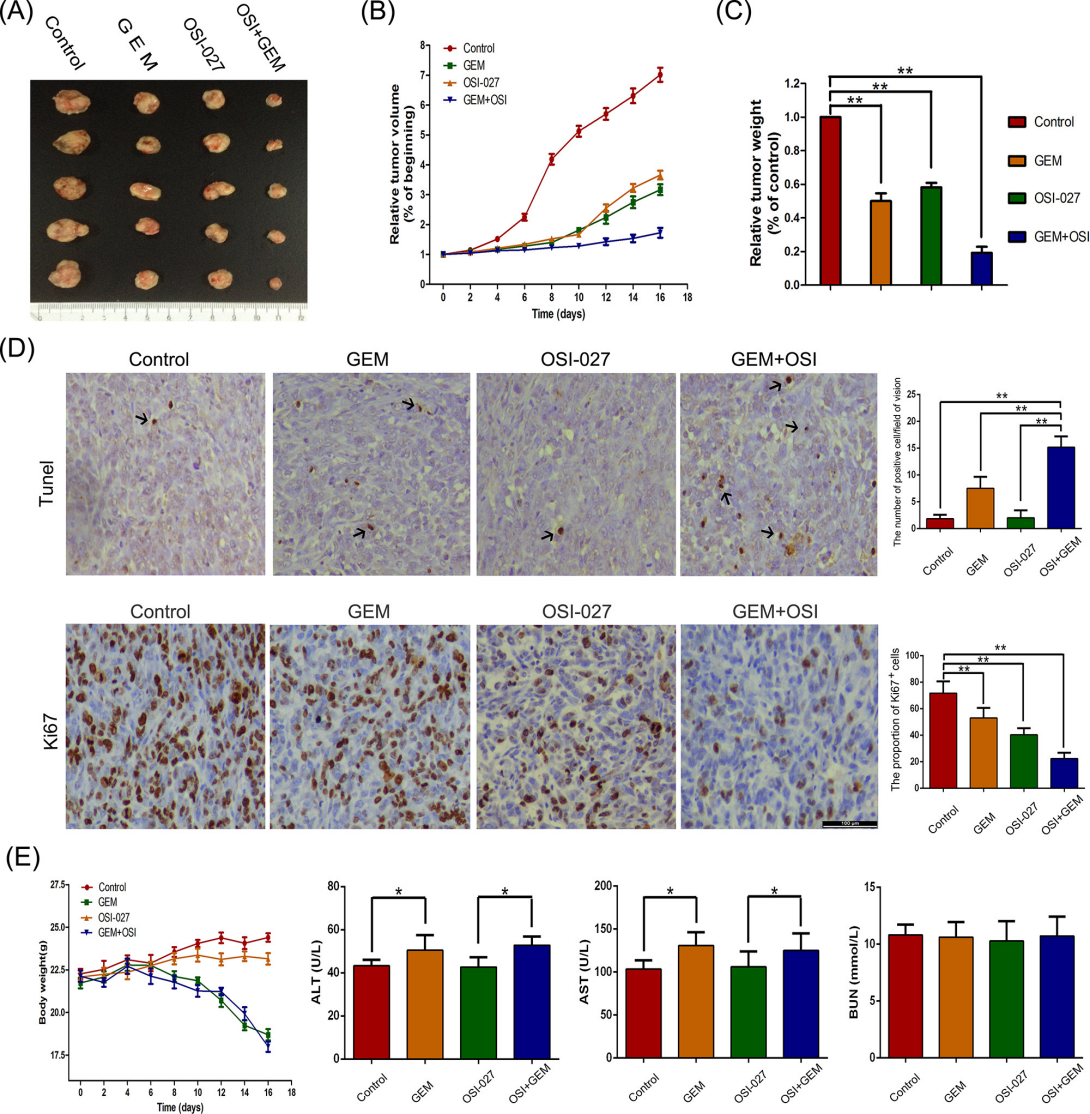
The effects of gemcitabine (GEM) and OSI-027 and their combination on tumor weight and volume in a pancreatic xenograft mouse model **A.** Resected tumors showing that combination therapy reduced tumor size greater than either therapy alone. **B.** Tumor volume over time indicating that combination therapy was more effective than either treatment alone. **C.** Tumor weight showing that every therapy combination significantly reduced weight relative to controls (**P < 0.01). **D.** TUNEL assay showing OSI-027 enhanced the apoptosis induced by GEM; Ki67 staining revealing both OSI-027 and GEM inhibited the proliferation of xenograft tumor cells and combined treatment with OSI-027 and GEM showed a greater capacity to inhibit proliferation (**P < 0.01). **E.** The body weight of mice did not significantly decrease when GEM was combined with OSI-027 compared to GEM mono-therapy. OSI-027 had no effects on AST or ALT and did not increase the AST and ALT raise induced by GEM (*P < 0.05). Both OSI-027 and GEM had no effects on BUN.

In the Panc-1 xenograft nude mouse model, both OSI-027 and GEM reduced tumor volume (control: 0.99 ± 0.2 m^3^; GEM: 0.63 ± 0.09 m^3^; OSI-027: 0.67 ± 0.13 m^3^) and weight (control: 1.5 ± 0.3 g; GEM: 0.95 ± 0.13 g; OSI-027: 1.01 ± 0.19 g). The combination therapy consisting of OSI-027 and GEM induced a significant reduction in tumor volume (0.16 ± 0.06 m^3^) and weight (0.24 ± 0.09 g) compared to either treatment alone (Figure 6A–6C). Terminal deoxynucleotidyl transferase dUTP nick end labeling (TUNEL) assay showed OSI-027 could not induce PDAC cells apoptosis; however, it enhanced the apoptosis induced by GEM. Immunohistochemical assay showed that both OSI-027 and GEM could inhibited the proliferation of PDAC cells compared to control, and combined treatment with OSI-027 and GEM showed a greater capacity to proliferation inhibition than mono-therapy (Figure 6D). Moreover, OSI-027 mono-therapy had little effect on the body weight of experimental mice and did not increase the body weight loss induced by GEM. Besides the influence on body weight, OSI-027 had no effect on the AST and ALT in nude mouse blood samples and did not increase the AST and ALT raise induced by GEM. Neither mono-therapy nor combined therapy had effects on BUN (Figure 6E).

## DISCUSSION

PDAC is a deadly disease with limited treatment options. Moreover, surgical tumor removal, heralded as the most effective treatment, is suitable in less than 20% of patients [5]. Although there has been some progress with respect to the development of novel, targeted therapies in PDAC, such as GEM, more therapeutics are desperately needed. One such potential molecule, OSI-027 has been shown to inhibit mTORC1 and mTORC2, which are aberrantly activated in the vast majority of pancreatic tumors [9, 10]. Moreover, OSI-027 has shown promise for the treatment of several malignancies, including head and neck, bladder, and breast cancer [21, 27, 28]. Therefore, we investigated, for the first time, the *in vitro* and *in vitro* effects of OSI-027 in pancreatic cell lines and in a PDAC xenograft model.

Herein, we showed that OSI-027 was more effective than RAPA, which is known to only inhibit mTORC1, at inhibiting the proliferation of three distinct pancreatic cell lines. Moreover, studies have shown that greater RAPA sensitivity in cell lines results in greater Akt activation, thereby attenuating treatment efficacy [29]. Thus, we further investigated the implications of OSI-027 treatment on downstream Akt effectors. As a consequence of blocking mTORC1 and mTORC2, unlike RAPA that only blocks mTORC1, cell cycle regulators and downstream effectors of Akt activation, including cyclin D1 and CDK4, were downregulated. Thus, the two-pronged mTOR approach with OSI-027 appears more beneficial than only targeting mTORC1 with RAPA in PDAC. Evidence from other cancers support this theory, as mTORC2 inhibition has been shown to be effective for inhibiting colon cancer cell proliferation [30]. Moreover, in leukemia, targeting both mTORC1 and mTORC2 exhibited significant suppressive effects, decreases Akt activation, and induces apoptosis [20, 31, 32].

We also investigated the effects of OSI-027 combination therapy with the first-line chemotherapeutic agent GEM; *in vitro* and *in vivo* experiments both showed synergistic effects. *In vitro*, OSI-027 downregulated MDR1, which has been shown to suppress apoptotic stimuli [33]. Thus, we believe that OSI-027 enhanced the killing effect of GEM by downregulating MDR-1 expression. Moreover, although most pancreatic tumors initially show sensitivity to GEM therapy, patients quickly become resistant to this treatment [34], in which MDR-1 is typically implicated [35]. Thus, in addition to increasing the effectiveness of the cell killing effect of GEM, OSI-027 may also delay drug resistance, a hypothesis that requires further investigation. In head and neck cancer, it was shown that inhibiting both mTORC1 and mTORC1 increased sensitivity to epidermal growth factor inhibitors; thus, we believe that OSI-027 should be used with other chemotherapy agents, such as GEM, to increase their efficacy [21].

In a xenograft mouse model using Panc-1 cells, the combination treatment of OSI-027 with GEM significantly reduced tumor weight and volume relative to either treatment as a mono-therapy. As a common side-effect of GEM therapy is difficulty eating and weight loss, we were further encouraged by the lack of additional weight loss when GEM was combined with OSI-027.

This study is not without several limitations. For instance, in order to determine the effects of mTORC2 on cell cycle, we only downregulated its expression using OSI-027; it remains to be seen whether upregulation of mTORC2 has a positive effect on cell proliferation. Secondly, we only observed the relationship between phospho-Akt and MDR1; more in depth analyses are required to further elucidate the implications and mediators of this interaction.

In this study, we explored the important roles of mTORC1 mTORC2 in the proliferation of PDAC cells, and provided evidence of the efficacy of a new compound, OSI-027, in PDAC. Moreover, downregulating mTORC1/mTORC2 may also reduce drug resistance in PDAC, which is a common occurrence in the clinic. Finally, OSI-027 combined with GEM showed synergistic killing action, suggesting that it might be a potent combination in the fight against PDAC and should be tested in further studies.

## MATERIALS AND METHODS

### Cell culture

Human PDAC cell lines (Panc-1, BxPC-3, CFPAC-1) were obtained from the Shanghai Institute for Biological Science (Shanghai, the People's Republic of China) and were cultured using standard techniques. Panc-1 and CFPAC-1 were cultured in high glucose DMEM (Gibco, Carlsbad, CA, USA) supplemented with 10% fetal bovine serum (FBS; Gibco) and 1% penicillin/streptomycin (Sigma-Aldrich, St. Louis, MO, USA). BxPC-3 was cultured in RPMI-1640 (Gibco) supplemented with 10% FBS and 1% penicillin/streptomycin. Cells were maintained at 37°C in a humidified incubator with 5% CO2 and were used within 3 months of thawing. Cells were monitored weekly by phase contrast microscopy to ensure that they were in logarithmic growth phase.

### Effects of OSI-027 and RAPA on PDAC cells

Prior to experimentation, cells were plated at a density of 3000 cells per well in 96-well plates and the media was removed and replaced by serum free media; 24 hours later the serum free media was replaced by complete media with the addition of OSI-027 (0, 1.25, 2.5, 5, 10, 20, 40, and 80 μM; Selleck Chemicals, Houston, TX, USA) or RAPA (0, 1.25, 2.5, 5, 10, 20, 40, and 80 μM; Sigma-Aldrich, St. Louis, MO, USA). Using the Cell Counting Kit (CCK)-8 (KeyGEN BioTech, Nanjing, People's Republic of China), we determined cytotoxicity and the half maximal inhibitory concentration (IC50) at 24 hours. In order to detect the cytotoxicity of OSI-027 and RAPA at different time points, PDAC cells were cultured as previously described, and the serum free media was replaced by complete media with the addition of OSI-027 (10 μM) and RAPA (100 nM), the cytotoxicity of OSI-027 and RAPA was tested at 24, 48, and 72 hours after drug treatment. Cell proliferation was determined using different concentrations of OSI-027 (5 and 10 μM in Panc-1, BxPC-3, and CFPAC-1) and RAPA (100 nM) with the Cell-light EdU Apollo 488 *in vitro* kit (RIBOBIO, Guangzhou, People's Republic of China) as per the manufacturer's instructions.

### Effects of OSI-027 and RAPA on cell cycle

In order to determine the effects of OSI-027 and RAPA on cell cycle, we performed flow cytometry. Briefly, PDAC cells were treated with OSI-027 (Panc-1, BxPC-3, and CFPAC-1: 5 and 10 μM) and RAPA (100 nM), 24 hours later cells were washed three times with ice-cold PBS, DNA was stained with propidium iodide (PI; Dawen, Shanghai, China) and flow cytometry. Quantitative cell cycle analysis was conducted using ModFit software (Verity Software House, Middlesham, ME, USA). Cell proliferation was expressed as the percentage of S + G2/M phase cells.

### Effects of OSI-027 and RAPA on mTOR pathway-related proteins

Western-blot was used to determine the effects of OSI-027 (Panc-1, BxPC-3 and CFPAC-1: 5 and 10 μM) and RAPA (100 nM) on mTOR pathway proteins. Western-blot was performed in accordance with standard protocols. Briefly, 40 μg proteins of cell lysates were separated by 10% sodium dodecyl sulfate–polyacrylamide gel electrophoresis and were transferred to polyvinylidene difluoride membranes. The membranes were blocked with Tris-buffered saline/0.1% Tween 20 containing 5% bovine serum albumin and were incubated with specific primary antibodies (all 1:1, 000 dilution). Six hours later, membranes were washed (3 times; 30 minutes) with Tris-buffered saline/0.1% Tween 20 followed by incubated with appropriate horseradish peroxidase (HRP)-conjugated secondary antibodies (all 1:2, 000 dilution). All antibodies were purchased from Cell Signaling Technology except anti-Cyclin D1 (Epitomics, Burlingame, CA, USA) and the HRP-conjugated secondary antibodies (Beyotime Institute of Biotechnology, Shanghai, People's Republic of China).

### mTOR knockdown in pancreatic cell lines

Using siRNA, the expression of mTOR, mTOR1 and mTOR2 was knocked down in PDAC cell lines. Transfections were performed using Lipofectamine 2000 (Invitrogen, Carlsbad, CA, USA) according to the manufacturer's protocol. Six hours later, the transfection medium was replaced by complete medium. mTOR siRNA (GenePharma, Shanghai, China), mTOR1 siRNA (GenePharma, Shanghai, China), mTOR2 siRNA (GenePharma, Shanghai, China) and negative-control siRNA (GenePharma, Shanghai, China) were transfected into cells at a concentration of 100 nM. The efficiency of siRNA interference was evaluated using Western blot as previous described.

### OSI-027 and GEM

Next, we tested the cytotoxicity of OSI-027 (0, 1.25, 2.5, 5, 10, 20, 40, 80 μM), GEM (0, 0.125, 0.25, 0.5, 1, 2, 4, 8 μM) and the combination of OSI-027 and GEM in the three PDAC cell lines to determine if they had a synergistic effect according to combination index (CI; CI = (IC_50_)_C1_/(IC_50_)_S or P_+(IC_50_)_C2_/(IC_50_)_D_. Where CI ≤ 0.9, > 0.9 and < 1.1, ≥ 1.1 indicate synergism, additive effect, and antagonistic effect, respectively), which was introduced by Chou and Talalay [36]. We also used flow cytometry to assess apoptosis; PDAC cell lines were treated with GEM (Panc-1: 40 μM; BxPC-3 and CFPAC-1: 1 μM), OSI-027 (Panc-1: 20 μM; BxPC-3 and CFPAC-1: 10 μM) and their combination, 24 hours later cells were harvested and washed twice with ice-cold PBS, stained by Annexin V-PE/7AAD apoptosis kit (BD Biosciences, Franklin Lakes, NJ, USA) according to the manufacturer's introduction, and analyzed by a BD FACS Caliber flow cytometer using BD Cell Quest software.

### OSI-027 and multi-drug resistance 1

As multi-drug resistance protein (MDR) 1 has been implicated in resistance development following GEM therapy in PDAC, we treated cells with OSI-027 (Panc-1: 20 μM; BxPC-3 and CFPAC-1: 10 μM) and then measured MDR1 (Cell Signaling Technology) levels by Western blot as previously described.

As the Akt pathway has been shown to regulate MDR1 activity [37], we knocked down Akt expression in these same cells using Akt siRNA (GenePharma, Shanghai, China), and observed MDR1 changes in order to determine if OSI-027 regulated MDR1 expression through Akt. Moreover, we assessed MDR1 levels via Western blot in cells that were treated with the following drug combinations: OSI-027 (Panc-1: 20 μM; BxPC-3 and CFPAC-1: 10 μM) + cycloheximide (CHX; 1 μM; Sigma-Aldrich); OSI-027 (Panc-1: 20 μM; BxPC-3 and CFPAC-1: 10 μM) + MG-132 (100 μg/mL; Sigma-Aldrich); MG-132 mono-therapy; and CHX monotherapy. As with Akt, CHX (synthesis inhibition) and MG-132 (protein degradation) were selected to determine if OSI-027 suppressed MDR1 via inhibiting its synthesis.

In order to confirm that OSI-027 enhanced the sensitivity of PDAC to GEM was through inhibiting MDR1 levels, we knocked down MDR1 expression using siRNA, and determined the effects of this treatment on genetic and protein levels. We also determined the IC50 of the OSI-027 + GEM combination (0, 1.25, 2.5, 5, 10, 20, 40, 80 μM; and 0, 0.125, 0.25, 0.5, 1, 2, 4, 8 μM, respectively) on cell lines that had previously been treated with MDR1 siRNA using CCK-8 as per the manufacturer's instructions.

### Quantitative reverse transcription–PCR

Cells were treated with vehicle or OSI-027 (Panc-1, BxPC-3 and CFPAC-1: 10 and 20 μM) for 24 hours or interfered using MDR1 siRNA as described in section 5. Total RNA was extracted and reverse transcribed using TRIzol Reagents (Invitrogen, Shanghai, China) and Prime Script reagent RT Kit (Takara Biotechnology, Dalian, China) according to the manufacturer's protocol. Primers for MDR1 were designed and obtained from Takara (forward, 5′-TGACTCAGGAGCAGAAGTTTGAACA-3′; reverse, 5′-AAATACATCATTGCCTGGGTGAAG-3′). Real-time qPCR was performed on the ABI Prism 7900HT Real-Time System (Applied Biosystems Inc, Shanghai, People's Republic of China), and MDR1 mRNA expression level was normalized to that of β-actin (forward, 5′-TGGCACCCAGCACAATGAA-3′; reverse, 5′-CTAAGTCATAGTCCGCCTAGAAGCA-3′).

### *In vivo* model

Human pancreatic cells (dissolved in PBS, 2 × 10^7^/100 μM, 100 μM/per nude mice; Panc-1) were injected subcutaneously in the right flank of BALB/c nude mice. After tumor volume reached 150–200 mm^3^, 24 tumor-bearing mice were randomly divided into four groups (control group, GEM group, OSI-027 group, and combination group). The GEM group was injected with GEM (25 mg/kg/d; dissolved in PBS) via caudal vein; OSI-027 group was treated with OSI-027 (30 mg/kg/d; dissolved in 20% Trappsol [CTD Holdings Inc, Alachua, FL, USA]) orally; the combination group was treated with GEM (25 mg/kg/d) and OSI-027 (30 mg/kg/d) as previously described; and the control group was injected with equal quantity of vehicle via caudal vein. Changes in mouse body weight were monitored throughout the study, tumor growth was observed every 2 days and was calculated by the formula: tumor volume = length × width × height/2. Mice were sacrificed at day 16, tumors were excised, weighed, and volume was measured for between group comparisons. Finally, 0.5 mL of blood was obtained from each nude mouse by cardiac puncture and send to clinical laboratory to detect liver and kidney function.

### Terminal deoxynucleotidyl transferase dUTP nick end labeling (TUNEL) assay and immunohistochemical assay

Tumor tissues were fixed in 10% formalin before embedded in paraffin and4 μm thick sections were prepared. The In Situ Cell Death Detection Kit, POD (Roche Molecular Biochemicals, Indianapolis, IN, USA) was used to detect cellular apoptosis in tumor tissue according to the manufacturer's instructions. Cells with brown stained were considered apoptotic cells. For immunohistochemistry staining, the slides were incubated with human anti-Ki67 (Sigma-aldrich, USA) (1:400) antibody followed by incubated with horseradish peroxidase-conjugated antibodies against rabbit immunoglobulin using Histostain-Plus Kit (Haoran-Bio, Shanghai, China), Finally, the sections were counterstained with hematoxylin and incubated the negative controls with PBS instead of the specific primary antibody. Five sections per tumor tissue were examined.

### Statistical analysis

All experiments were repeated at least three times and data are presented as means and standard deviation (SD) values. Prism5 (GraphPad, SanDiego, CA, USA) was use to perform Statistical analysis. Statistical significance was defined as *P*-value < 0.05 using Student's *t*-test between different groups.

## Abbreviations

PDAC: pancreatic ductal adenocarcinoma
RAPA: rapamycin
mTOR: mammalian target of rapamycin
mTORC1: mTOR complex 1
MDR1: multidrug resistance protein 1
GEM: gemcitabine
5-FU: Fluorouracil
CI: combination index
IC_50_: half maximal inhibitory concentration
CCK: Cell Counting Kit
PI3K: phosphatidylinositol 3′-kinase
MEK: mitogen-activated protein kinase
CDK4: cyclin-dependent kinase 4
